# Safety and Efficacy of Nemolizumab for Patients with Pruritus: A Systematic Review and Meta-Regression Analysis of Randomized Controlled Trial

**DOI:** 10.3389/fimmu.2022.825312

**Published:** 2022-04-26

**Authors:** Junqin Liang, Fengxia Hu, Maoli Dan, Yingbing Sang, Kailibinuer Abulikemu, Qian Wang, Yongzhen Hong, Xiaojing Kang

**Affiliations:** Department of Dermatology and Venereology, People’s Hospital of Xinjiang Uygur Autonomous Region, Xinjiang Clinical Research Center for Dermatologic Diseases, Xinjiang Key Laboratory of Dermatology Research (XJYS1707), Urumqi, China

**Keywords:** pruritus, atopic dermatitis (AD), nemolizumab, randomized controlled trial, meta-analysis

## Abstract

**Background:**

Nemolizumab is deemed as a promising drug for atopic dermatitis (AD) patients with pruritus.

**Objective:**

This study aimed to evaluate the efficacy of nemolizumab in treating patients with AD and the association between the dosage or regimen of nemolizumab with the improvement in clinical indices.

**Methods and Materials:**

PubMed, Embase, and the Cochrane Library were searched for randomized controlled trials (RCTs) published from inception to August 2021.

**Results:**

A total of 14 cohorts of participants from six randomized controlled studies were included in the meta-analysis. Nemolizumab significantly reduced the pruritus VAS (WMD = −18.86, 95% CI: −27.57 to −10.15, *p* < 0.001; *I* ^2^ = 56.2%, *p*_heterogeneity_ = 0.005) and EASI (WMD = −11.76, 95% CI: −20.55 to −2.96, *p* = 0.009; *I* ^2^ = 0%, *p*_heterogeneity_ = 0.978) scores compared with placebo. No significant difference was observed in the occurrence of any AEs (RR = 1.03, 95% CI: 0.93 to 1.13, *p* = 0.593; *I* ^2^ = 0%, *p*_heterogeneity_ = 0.980) between the two groups. The univariate meta-regression showed that both the dosage and study duration had no association with the change of pruritus VAS score.

**Conclusion:**

Nemolizumab presented a promising effect based on the difference in the average change in pruritus VAS and EASI scores compared with placebo. The results indicated its efficacy in relieving pruritus and the severity of AD and improving patients’ quality of life.

## Introduction

Atopic dermatitis (AD) is a chronic inflammatory skin disease with a current worldwide prevalence of 5%–25% (1). Moderate-to-severe AD is often refractory to first-line topical treatments, while systemic immunosuppressants are commonly used in clinical treatment (2). However, the use of traditional systemic treatments including systemic corticosteroids, phototherapy, and immunosuppressants is limited by the safety risk (3). Due to the undesirable adverse effects (AEs) of traditional systemic treatment, there is still an unmet need for safe and effective long-term therapy for moderate-to-severe AD. The symptoms of AD are characterized by pruritus and skin barrier dysfunction, which are triggered by an immune response to antigenic substances and mechanical irritation (4–7). Some patients have pruritus even if other symptoms are well controlled by topical glucocorticoids and antihistamines, and their effects in AD are limited or associated with long-term side effects (8, 9). Thus, among patients with AD, the primary therapeutic objective should be to relieve pruritus, improve dermatitis, and enhance quality of life.

Interleukin-31 (IL-31) is believed to play a key role in the pathogenesis of AD and especially in the development of pruritus (10–13). Nemolizumab, a humanized monoclonal antibody, binds to IL-31 receptor A and inhibits IL-31 signaling in cells (14, 15). Some studies indicated that nemolizumab might reduce the symptom of pruritus with AD (16, 17). Therefore, nemolizumab is deemed as a promising drug for AD patients with pruritus. When nemolizumab is administered in a hospital, the body weight-normalized dose is not problematic. However, considering that an autoinjection situation might be warranted in the future, there is still no consensus on the flat-dose regimen of the utilization of nemolizumab. Although several previous clinical trials have attempted to investigate an appropriate self-injection regimen, the results remained inconclusive (15, 18).

Although a recently published meta-analysis demonstrated the efficacy of nemolizumab for patients with AD by pooling the results from four randomized controlled trials (RCTs), it failed to assess the effect of dosage or the times of injections on the study outcomes (19). Moreover, several additional RCTs implemented with different doses or times of injections have been published recently. Therefore, this meta-analysis aimed to evaluate the efficacy of nemolizumab in treating patients with AD by assessing the amelioration of pruritus as well as to investigate the association between dosage or regimen of nemolizumab with the improvement in clinical indices.

## Materials and Methods

### Literature Search

This meta-analysis was performed according to the Preferred Reporting Items for Systematic Reviews and Meta-Analyses (PRISMA) guidelines (20). This study does not contain any participants and ethics approval is not applicable. The literature search was performed based on the PICO principle (21). PubMed, Embase, and the Cochrane Library were searched for available papers published up to August 2021 for potentially eligible studies, using the MeSH terms “pruritus,” “dermatitis, atopic,” and “nemolizumab” and relevant keywords. The eligibility criteria were 1) population: patients with atopic dermatitis and pruritus, 2) intervention: nemolizumab, 3) control: placebo, 4) randomized controlled trial, and 5) full text in English.

### Quality Appraisal

The level of evidence of the included studies was assessed independently by two authors (FH and MD) according to version 2 of the Cochrane tool for assessing risk of bias in randomized trials (RoB 2) (22). The included studies were assessed respectively in five domains regarding the 1) bias arising from the randomization process, 2) bias due to deviations from intended interventions, 3) bias due to missing outcome data, 4) bias in the measurement of the outcome, and 5) bias in the selection of the reported result. Finally, an overall evaluation was concluded per study based on the assessments of each individual aspect. Any discrepancy in the assessment was resolved by discussion until a consensus was reached.

### Data Extraction

The study characteristics (authors, year of publication, country, study phase, study duration, sample size, gender, and age of the patients), treatment parameters (the regimen of the desired treatment and the dose of each injection), and outcomes were extracted by two authors (KA and YH) independently. Any discrepancy was resolved by discussion.

### Outcomes

Pruritus visual analog scale (VAS) is a commonly used scoring scale to evaluate the pruritus severity which ranged from 0 to 100 with higher scores indicating worse pruritus (23). Eczema Area and Severity Index (EASI) (24) and AEs were also extracted as the study outcomes. The primary outcome in the present meta-analysis was the mean change in pruritus VAS score from baseline to the end of the study. The secondary outcomes were mean change in EASI score from baseline to the end of the study and the occurrence of any AEs.

### Data Synthesis

This study was designed to directly compare the treatment of nemolizumab with placebo. Some included studies contained more than one treatment arm to compare the effects of different doses of nemolizumab. In such instances, we split the participants into several cohorts of patients to directly compare the efficacy of each dosage of nemolizumab with placebo. To avoid arbitrary omission of relevant groups and double counting of participants, we included each pairwise comparison separately, but with the placebo group divided out approximately evenly among the comparisons (25). Pruritus VAS and EASI at the last follow-up were extracted for the nemolizumab and placebo groups, respectively. If the results were not presented as means and standard deviations, the means and standard deviations were estimated based on the reported parameters (median, range, or standard error) (26).

### Statistical Analysis

All analyses were performed using STATA SE 14.0 (StataCorp, College Station, TX, USA). Effects [weighted mean difference (WMD) or standardized mean difference (SMD), as appropriate] and the corresponding 95% confidence intervals (CIs) were used to compare the outcomes. Statistical heterogeneity among the studies was evaluated using Cochran’s *Q*-test and the *I* ^2^ index. *I* ^2^ >50% and *Q*-test *p <*0.10 indicated high heterogeneity, and the random-effects model was used; otherwise, the fixed-effects model was applied. *p <*0.05 was considered statistically significant. The odds ratio (OR) was used to assess the differences in the number of AEs for each group, in which case the funnel plots and Egger’s test can yield misleading results (27). According to the Cochrane Handbook, the publication bias was analyzed only when the number of cohorts included in each meta-analysis was more than 10. Funnel plots, Begg’s test, and Egger’s test were conducted respectively to assess the potential publication bias. Subgroup analysis and meta-regression were applied to analyze the impact of covariates on the study outcomes.

## Results

### Selection of the Studies

**Figure 1:**
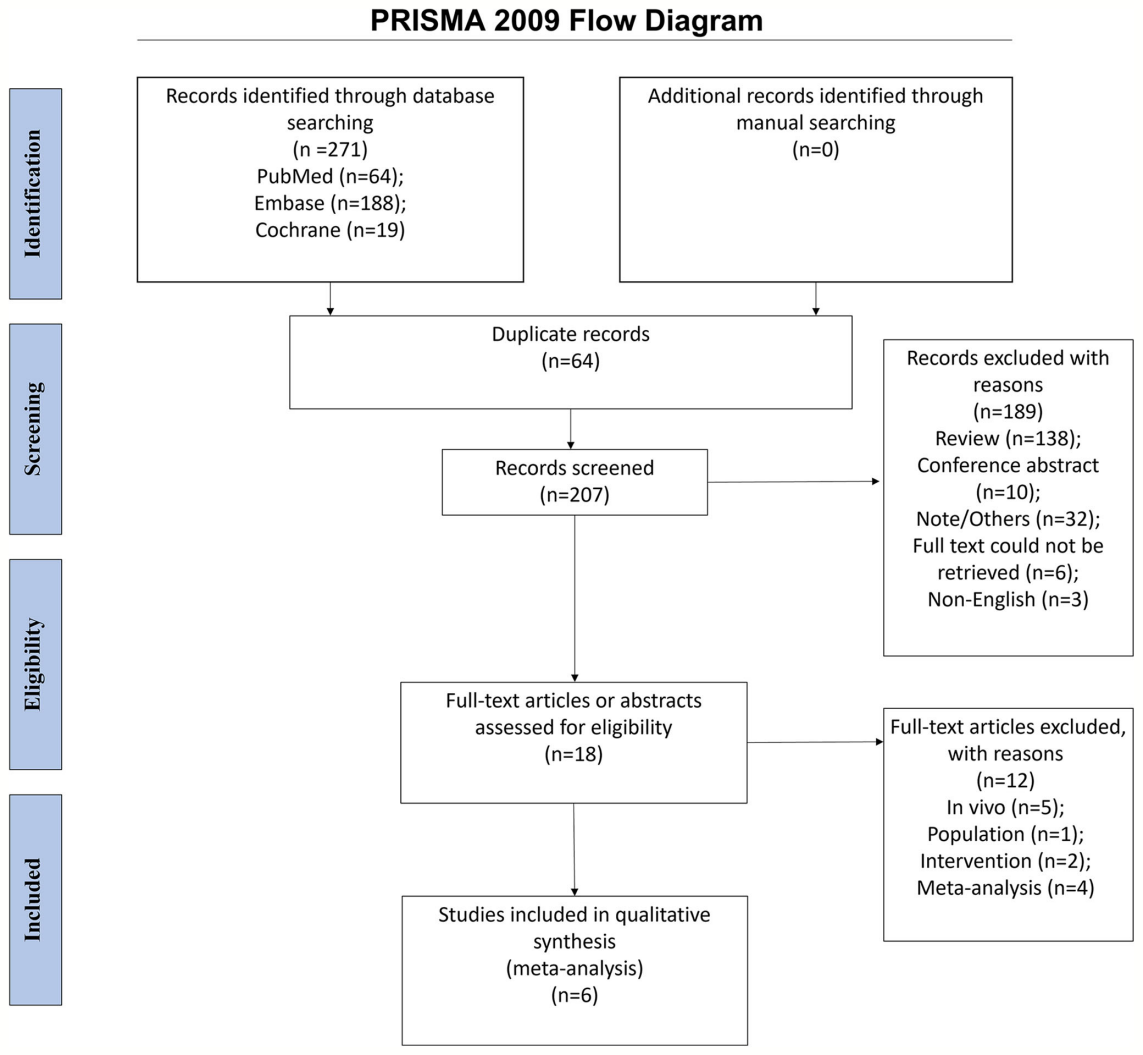
PRISMA 2009 flow diagram.

The initial search yielded 271 entries. After removing the duplicates, 207 records were screened, and 189 were excluded. Eighteen full-text papers were assessed for eligibility, and 12 were excluded (*in-vivo* study, *n* = 5; population, *n* = 1; intervention, *n* = 2; meta-analyses, *n* = 4). Finally, 14 cohorts of participants consisting of six randomized controlled studies were included in the meta-analysis (**Figure 1**).

### Characteristics of the studies

This meta-analysis finally included 569 and 240 patients who received nemolizumab and placebo, respectively. Of the six included studies, there were one phase I trial (15), four phase II trials (16, 18, 28, 29), and one phase III trial (17). The time from treatment to outcome assessment ranged from 4 to 24 weeks, and the times of total injections varied from single injection (in two trials), three times (in two trials) to four times (in two trials). Details of the included studies are summarized in **Table 1**.

**Table 1:** Characteristics of the included studies.

Author, year (ClinicalTrial NO.)	Country	Phase	Study duration	Treatment arms	Dose	Sample size		Male, %		Age
						Intervention	Control	Intervention	Control	
Kabashima, 2020 (not available)	Japan	3	16-week	4 Q4W	2.0 mg/kg	143	72	65	67	13+
Kinugasa, 2021a (JAPIC: JapicCTI-15296)*	Japan	2	4-week	Single injection	0.125 mg/kg	14	5	71.4	\	20+
Kinugasa, 2021b (JAPIC: JapicCTI-15296)*	Japan	2	4-week	Single injection	0.5 mg/kg	13	4	69.2	\	20+
Kinugasa, 2021c (JAPIC: JapicCTI-15296)*	Japan	2	4-week	Single injection	2.0 mg/kg	14	5	100	0	20+
Nemoto, 2016a (not available)	Japan	1/1b	8-week	Single injection	0.3 mg/kg	9	3	88.9	\	20-49
Nemoto, 2016b (not available)	Japan	1/1b	8-week	Single injection	1.0 mg/kg	9	3	66.7	\	20-49
Nemoto, 2016c (not available)	Japan	1/1b	8-week	Single injection	3.0 mg/kg	9	3	77.8	\	20-49
Ruzicka, 2017a (NCT01986933)	Multinational	2	12-week	3 Q4W	0.1 mg/kg	53	18	53	\	18-65
Ruzicka, 2017b (NCT01986933)	Multinational	2	12-week	3 Q4W	0.5 mg/kg	54	18	41	\	18-65
Ruzicka, 2017c (NCT01986933)	Multinational	2	12-week	3 Q4W	2.0 mg/kg	52	17	60	\	18-65
Silverberg, 2020a (NCT03100344)	Multinational	2b	24-week	4 Q4W	0.136 mg/kg	55	19	52.7	54.4	18+
Silverberg, 2020b (NCT03100344)	Multinational	2b	24-week	4 Q4W	0.390 mg/kg	55	19	50.9	54.4	18+
Silverberg, 2020c (NCT03100344)	Multinational	2b	24-week	4 Q4W	1.118 mg/kg	55	18	45.6	54.4	18+
Stander, 2020 (NCT03181503)*	Multinational	2	18-week	3 Q4W	0.5 mg/kg	34	36	44	39	18+

*These studies were conducted among non-AD patients.

### Assessment for Risks of Bias

All trials in this meta-analysis were evaluated as low risk of bias. Only two studies (18, 28) were graded “some concerns” in the domain of bias arising from the randomization process owing to the absence of details in the allocation sequence (**Supplementary Table 1**).

### Pruritus Visual Analog Scale

**Figure 2:**
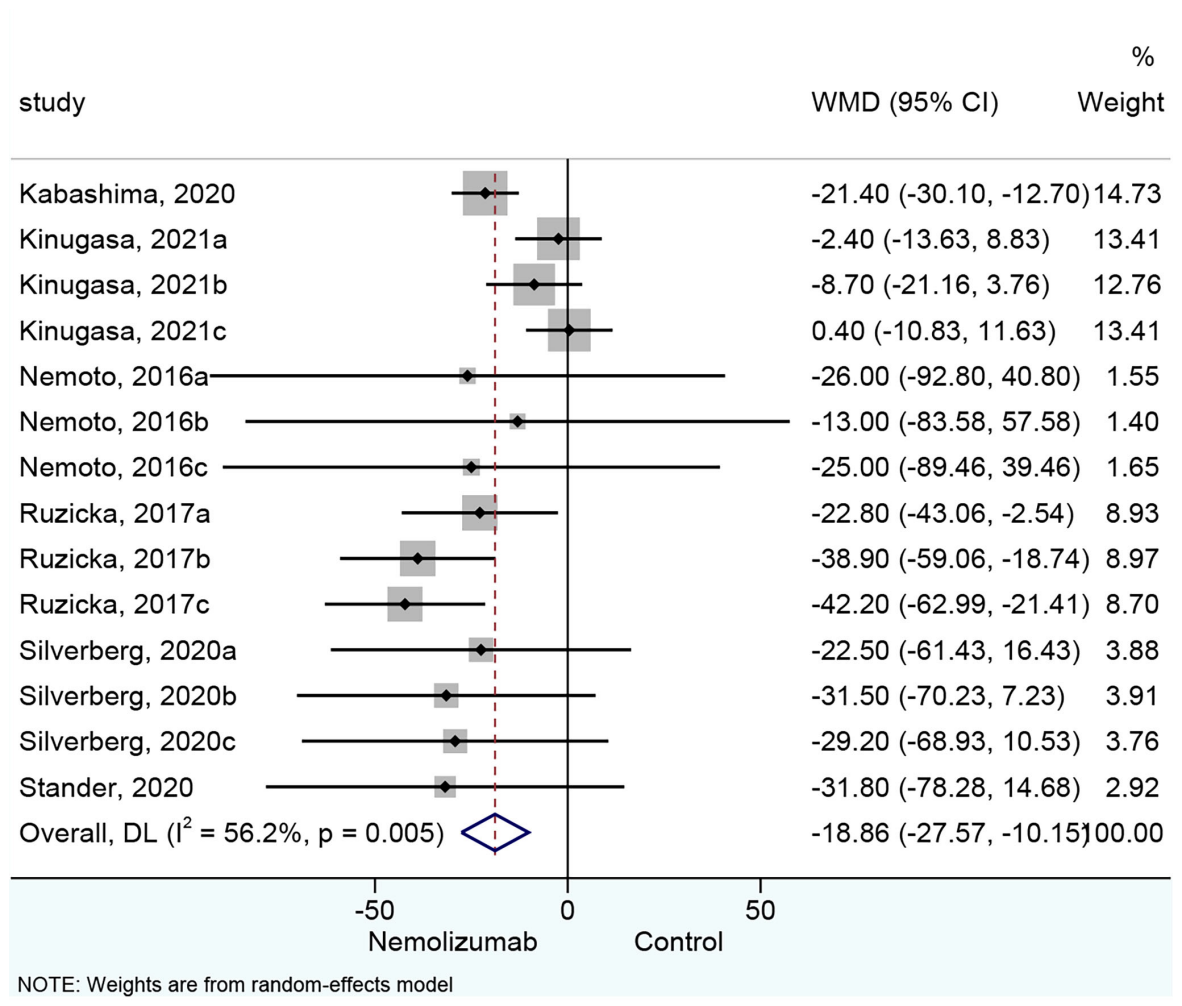
Forest plot of pruritus visual analog scale comparing nemolizumab and placebo.

All 14 cohorts of patients presented results about the changes in pruritus VAS score. Nemolizumab significantly reduced the pruritus VAS score compared with placebo (WMD = −18.86, 95% CI: −27.57 to −10.15, *p* < 0.001; *I* ^2^ = 56.2%, *p*_heterogeneity_ = 0.005, **Figure 2**). Six cohorts (15, 18) of patients reporting a single injection of nemolizumab during treatment revealed an insignificant reduction in pruritus VAS score compared with placebo (WMD = −3.76, 95% CI: −10.36 to 2.84, *p* = 0.264; *I* ^2^ = 0%, *p*_heterogeneity_ = 0.836, **Supplementary Figure 1A**), whereas the results of the subgroup analysis in the three times (WMD = −34.35, 95% CI: −45.77 to −22.94, *p* < 0.001; *I* ^2^ = 0%, *p*_heterogeneity_ = 0.572) and four times of injections (WMD = −22.22, 95% CI: −30.33 to −14.10, *p* < 0.001; *I* ^2^ = 0%, *p*_heterogeneity_ = 0.946) of nemolizumab showed a significant reduction in pruritus VAS score compared with placebo.

### Eczema Area and Severity Index Score

**Figure 3:**
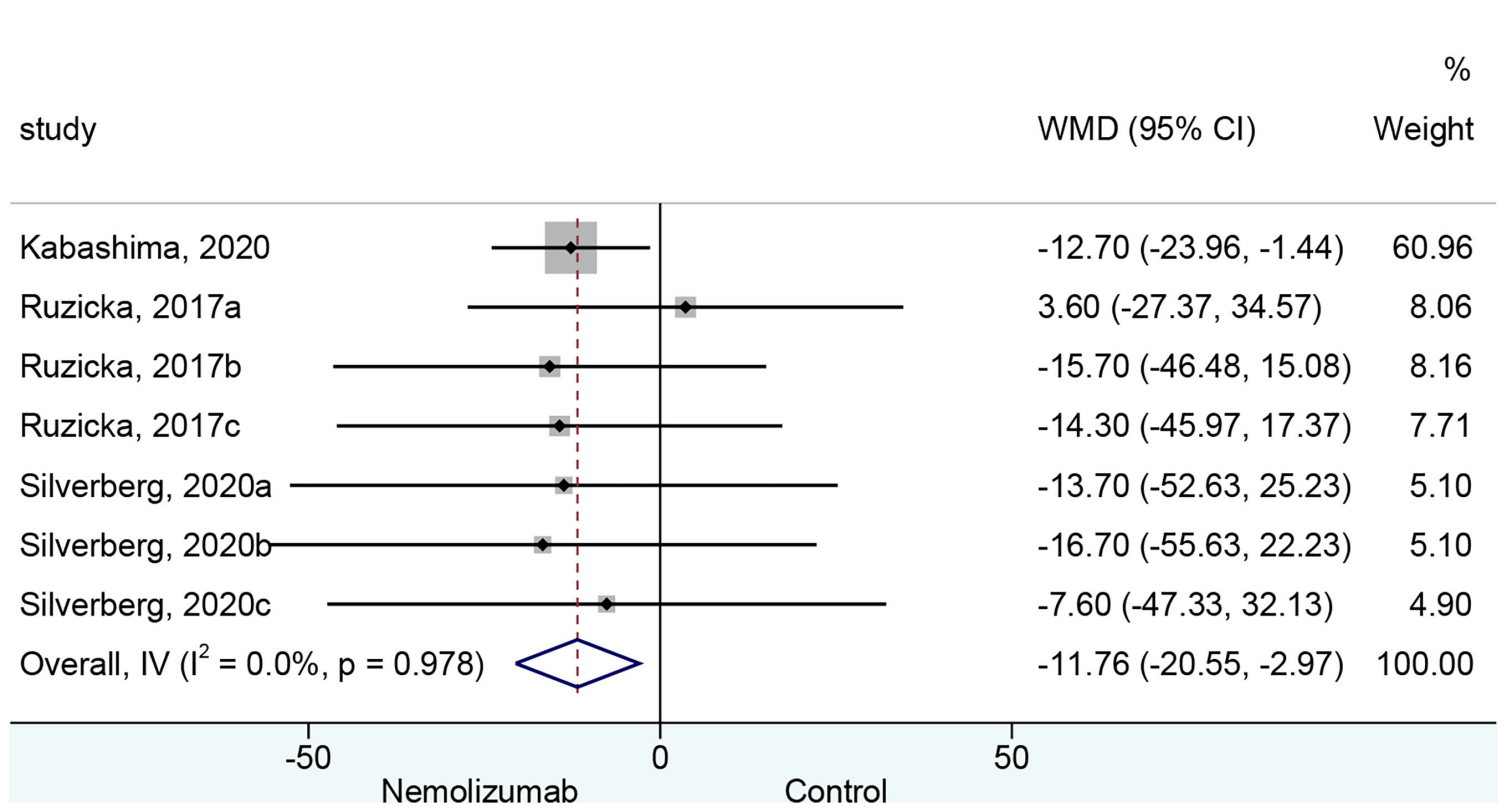
Forest plot of Eczema Area and Severity Index comparing nemolizumab and placebo.

Seven cohorts of patients retrieved from three studies reported the mean change of EASI score from baseline. There was a significant difference in the reduction of EASI score between nemolizumab and placebo (WMD = −11.76, 95% CI: −20.55 to −2.96, *p* = 0.009; *I* ^2^ = 0%, *p*_heterogeneity_ = 0.978, **Figure 3**). Subgroup analysis (**Table 2** and **Supplementary Figure 1B**) suggested no significant difference in the reduction of EASI score when there were only three injections of nemolizumab during treatment (WMD = −8.75, 95% CI: −26.72 to 9.22, *p* = 0.340; *I* ^2^ = 0%, *p*_heterogeneity_ = 0.630). However, significantly more reduction of EASI was observed in patients who were injected four times during the study period (WMD = −12.71, 95% CI: −22.79 to −2.62, *p* = 0.014; *I* ^2^ = 0%, *p*_heterogeneity_ = 0.991).

**Table 2:** Combined results of the subgroup analyses.

Variables	N	WMD (95%CI)	P (Heterogeneity)	I-square, %	P
Pruritus VAS	14	-18.86 (-27.57, -10.15)	0.005	56.2	<0.001
Pruritus VAS in AD	10	-26.05 (-32.63, -19.46)	0.804	0	<0.001
Single injection	6	-3.76 (-10.36, 2.84)	0.836	0	0.264
3 injections	4	-34.35 (-45.77, -22.94)	0.572	0	<0.001
4 injections	4	-22.22 (-30.33, -14.10)	0.946	0	<0.001
EASI	7	-11.76 (-2 0.55, -2.96)	0.978	0	0.009
3 injections	3	-8.75 (-26.72, 9.22)	0.630	0	0.340
4 injections	4	-12.71 (-22.79, -2.62)	0.991	0	0.014
Variables	N	RR (95%CI)	P (Heterogeneity)	I-square, %	P
AE	11	1.03 (0.93, 1.13)	0.980	0	0.593
Single injection	3	0.84 (0.63, 1.13)	0.836	0	0.249
3 injections	4	1.04 (0.88, 1.25)	0.983	0	0.626
4 injections	4	1.05 (0.93, 1.18)	0.898	0	0.415

### Adverse Events

**Figure 4:**
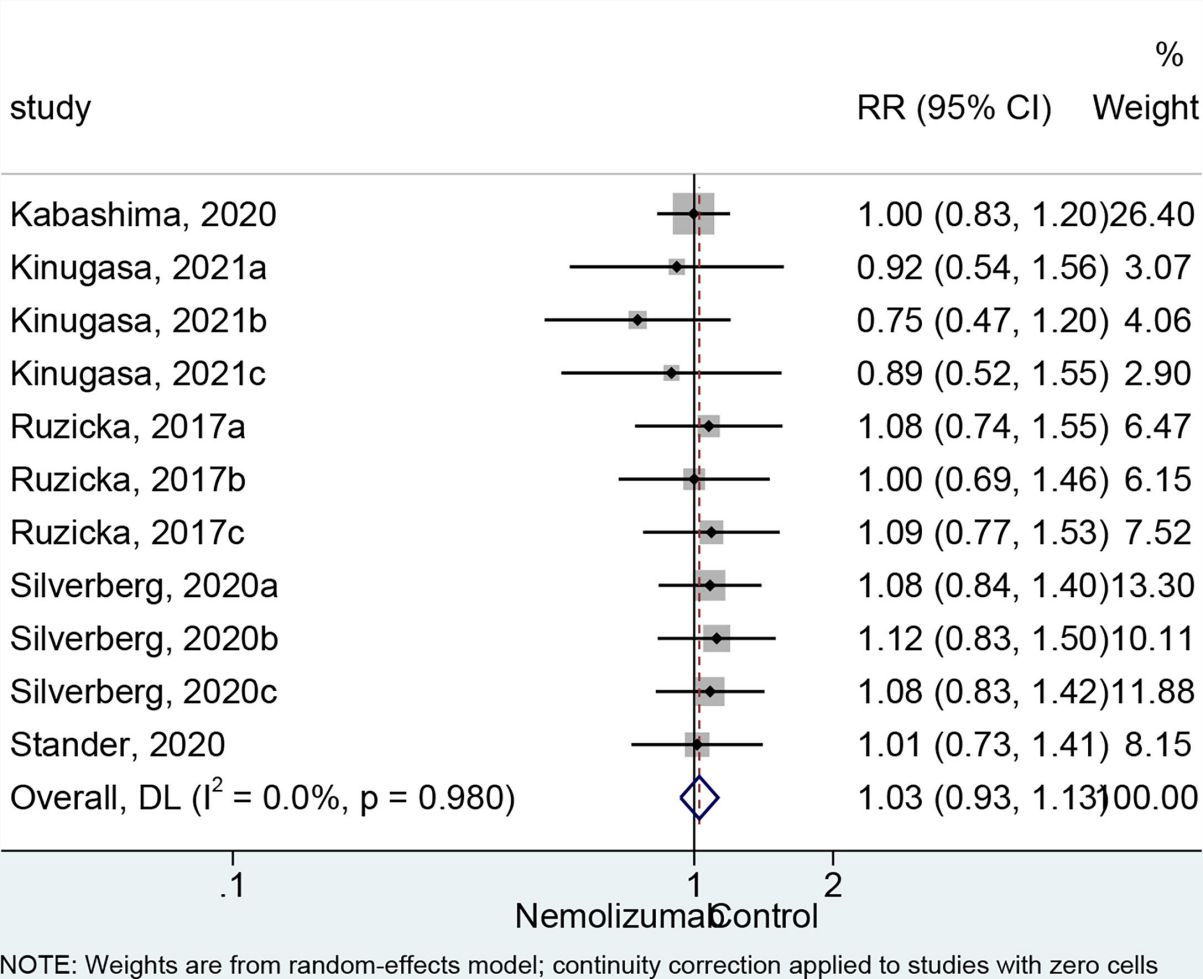
Forest plot of adverse events comparing nemolizumab and placebo.

There was no significant difference in the occurrence of any AEs between nemolizumab and placebo (RR = 1.03, 95% CI: 0.93 to 1.13, *p* = 0.593; *I* ^2^ = 0%, *p*_heterogeneity_ = 0.980, **Figure 4**). The comparable results were consistent regardless of the frequency of injection (**Supplementary Figure 1C**). The most frequently reported AEs from patients treated with nemolizumab were infections and skin or subcutaneous tissue disorders, including nasopharyngitis (occurred in 10%–32.7% of the patients) and exacerbated AD (occurred in 15%–28.1% of the patients). Several studies also reported that a few patients developed gastrointestinal disorders, respiratory disorders, thoracic disorders, mediastinal disorders, and administration site conditions after being treated with both nemolizumab and placebo, with relatively small and even percentile in the occurrences between groups (**Supplementary Table 2**). Besides, the severity of the AEs was generally mild, with only a few severe AEs being reported in both the nemolizumab and placebo groups (data not shown).

### Sensitivity Analysis, Publication Bias, and Meta-Regression

The results of the sensitivity analysis suggested that the results of the meta-analysis were robust (**Supplementary Figures 2A, B** **)**. No publication bias on pruritus VAS and AEs was detected according to the results of both Begg’s test and Egger’s test (**Supplementary Table 3**). The funnel plots are presented in **Supplementary Figures 3A, B**. Although no significant heterogeneity was observed between the included studies, meta-regression was performed to assess the possible effect of the dose of nemolizumab and length of study duration on the outcomes. The univariate meta-regression showed that both dosage (Beta = −0.61, 95% CI: −15.77 to 14.54, *p* = 0.931) and study duration (Beta = 2.04, 95% CI: −1.13 to 5.20, *p* = 0.186) had no association with the change of pruritus VAS score. This insignificant association was also observed in EASI and AE (**Supplementary Table 4**).

## Discussion

The results in this meta-analysis demonstrated the promising efficacy and safety of nemolizumab in treating patients with AD and pruritus. Compared with patients in the placebo group, the difference in the average change of pruritus VAS score was significant, indicating the efficacy of nemolizumab in relieving pruritus and in improving patients’ quality of life. In addition, the significantly greater reduction in the average change of EASI score comparing nemolizumab and placebo suggested a considerable remission in the severity of AD. Moreover, the comparable occurrences of any AEs in the treatment and placebo groups indicated a tolerable safety concern of nemolizumab for the treatment of AD. The results of our analysis are congruent with a previous meta-analysis, albeit differed a bit in the extent of reduction in the clinical indices. In the previous meta-analysis (19), compared with placebo, the average differences in the reduction of pruritus VAS score and EASI score from patients in the nemolizumab group are −3.96 (95% CI: −5.56 to −2.37, *p* < 0.001) and −0.31 (95% CI: −0.45 to −0.17, *p* < 0.001), respectively, and the relative risk of developing any AEs comparing nemolizumab and placebo was 0.84 (95% CI: 0.69 to 1.01, *p* = 0.069). It seems that the previous meta-analysis used a 10-point scale in pruritus VAS and EASI. Besides, the previous meta-analysis used the standardized mean difference to measure the pooled effect size. However, in this study, we used a 100-point scale to assess the differences in the change of pruritus VAS score and EASI score with weighted mean difference. Moreover, we included two newly published RCTs and had a total of six RCTs in this analysis. The variation in the extent of the results might be explained by the respective differences.

This meta-analysis also intended to evaluate how the differences in regimen would potentially affect the efficacy of nemolizumab in ameliorating the symptom of AD and pruritus. However, the exact total dose of nemolizumab was difficult to assess due to the limited information in each study, and the dose of each single injection varied significantly from 0.1 to 3.0 mg/kg. So, instead of calculating the total dose of nemolizumab during the treatment period, we performed a subgroup analysis on the total frequency of injections to estimate the effect of dosage. The results showed that the change of pruritus VAS score was not significantly different between the single injection of nemolizumab and placebo, and the occurrence of AEs was not different from placebo as well. A total of 3 times of injection could significantly outperform the placebo in the change of pruritus VAS score but not in the EASI score, and a total of 4 times of injection could yield a better reduction in both pruritus VAS and EASI compared with placebo. These results might indicate that a monthly total of 4 times of injections should be considered in future clinical designs. Although we failed to conclude the best dosage of nemolizumab in each injection, we did a meta-regression to analyze the association between the single dosage as well as the study duration with the differences in the change of pruritus VAS, EASI, and the occurrence of AEs. However, our results did not reveal a significant association between these two indicators.

An improved understanding of AD pathophysiology resulted in an explosion of studies to discover a novel and efficient agent for this population. Nemolizumab was developed as an inhibitor of IL-31 signaling, and several studies have presented findings that indicated a direct effect of IL-31 in the generation of pruritus in patients with atopic dermatitis (14, 15, 30, 31). Moreover, IL-31 might also promote the growth of sensory nerves or regulate the antimicrobial skin barrier (32, 33). Targeting interleukin-31 aims to decrease pruritus and signs of skin inflammation, which may result in the reduced severity of AD. Serum IL-31 concentrations in patients receiving maintenance hemodialysis were reported to be higher in those patients with pruritus than in those without pruritus (34). Kinugasa et al. also discovered a tendency for patients with higher serum IL-31 levels at baseline to have greater pruritus VAS reductions following nemolizumab treatment (18). Indeed, baseline IL-31 is a significant indicator that might influence the effect of nemolizumab. Future studies should focus on the role of IL-31 and its inhibition for the control of pruritus.

The present meta-analysis has limitations that must be considered when weighing the results. First, only six studies with a total of 809 patients were included in the analysis. Fortunately, all studies were sophisticatedly designed RCTs with low risk of bias, and the sensitivity analyses showed that the estimated parameters did not affect the conclusions. Besides, several studies did not report the parameters in a standard of mean and standard deviation. In such instances, reasonable estimates were used under the instruction of the Cochrane Handbook for systematic review.

In conclusion, the promising effect of nemolizumab reflected by the difference in the average change in pruritus VAS score and EASI compared with placebo indicated its efficacy in relieving pruritus and the severity of AD and improving patients’ quality of life. Moreover, the comparable occurrences of any AEs in the treatment and placebo groups indicated a tolerable safety concern of nemolizumab for the treatment of AD.

## Data Availability Statement

The original contributions presented in the study are included in the article/**Supplementary Material**. Further inquiries can be directed to the corresponding author.

## Author Contributions

JL contributed to the design of the study and drafting of the manuscript. FH and KA contributed to the data collection. MD and YH contributed to the literature research. YS and QW contributed to the partial drafting of the manuscript. XK contributed to the design of the study. All authors have read and approved the manuscript.

## Conflict of Interest

The authors declare that the research was conducted in the absence of any commercial or financial relationships that could be construed as a potential conflict of interest.

## Publisher’s Note

All claims expressed in this article are solely those of the authors and do not necessarily represent those of their affiliated organizations, or those of the publisher, the editors and the reviewers. Any product that may be evaluated in this article, or claim that may be made by its manufacturer, is not guaranteed or endorsed by the publisher.

## Supplementary Material

Supplementary Figure 1
**(A)** Forest plot of pruritus visual analog scale comparing nemolizumab and placebo by the frequency of injection. **(B)** Forest plot of Eczema Area and Severity Index comparing nemolizumab and placebo by the frequency of injection. **(C)** Forest plot of adverse events comparing nemolizumab and placebo by the frequency of injection.

Supplementary Figure 2
**(A)** Sensitivity analysis of pruritus visual analog scale. **(B)** Sensitivity analysis of Eczema Area and Severity Index. **(C)** Sensitivity analysis of adverse events

Supplementary Figure 3
**(A)** Funnel plots for pruritus visual analog scale. **(B)** Funnel plots for adverse events.

## Abbreviations

AD, atopic dermatitis; AEs, adverse effects; CIs, confidence intervals; EASI, Eczema Area and Severity Index; OR, odds ratio; RCTs, randomized controlled trials; RoB, risk of bias; SMD, standardized mean difference; VAS, visual analog scale; WMD, weighted mean difference.
