# Correction: Safety and efficacy of nemolizumab for patients with pruritus: a systematic review and meta-regression analysis of randomized controlled trial

**DOI:** 10.3389/fimmu.2026.1773876

**Published:** 2026-02-03

**Authors:** Junqin Liang, Fengxia Hu, Maoli Dan, Yingbing Sang, Kailibinuer Abulikemu, Qian Wang, Yongzhen Hong, Xiaojing Kang

**Affiliations:** Department of Dermatology and Venereology, People’s Hospital of Xinjiang Uygur Autonomous Region, Xinjiang Clinical Research Center for Dermatologic Diseases, Xinjiang Key Laboratory of Dermatology Research (XJYS1707), Urumqi, China

**Keywords:** pruritus, atopic dermatitis (AD), nemolizumab, randomized controlled trial, meta-analysis

The title of this article was erroneously given as: “Safety and Efficacy of Nemolizumab for Atopic Dermatitis with Pruritus: A systematic review and meta-regression analysis of randomized controlled trial”. The correct title of the article is “Safety and efficacy of nemolizumab for patients with pruritus: a systematic review and meta-regression analysis of randomized controlled trial”.

There was a mistake in **Table 1** as published. There was no ClinicalTrial.com.cn registration number provided, and the description of the study population was incomplete. The corrected **Table 1** appears below.

**Table 1:** Characteristics of the included studies.

Author, year (ClinicalTrial NO.)	Country	Phase	Study duration	Treatment arms	Dose	Sample size		Male, %		Age
						Intervention	Control	Intervention	Control	
Kabashima, 2020 (not available)	Japan	3	16-week	4 Q4W	2.0 mg/kg	143	72	65	67	13+
Kinugasa, 2021a (JAPIC: JapicCTI-15296)*	Japan	2	4-week	Single injection	0.125 mg/kg	14	5	71.4	\	20+
Kinugasa, 2021b (JAPIC: JapicCTI-15296)*	Japan	2	4-week	Single injection	0.5 mg/kg	13	4	69.2	\	20+
Kinugasa, 2021c (JAPIC: JapicCTI-15296)*	Japan	2	4-week	Single injection	2.0 mg/kg	14	5	100	0	20+
Nemoto, 2016a (not available)	Japan	1/1b	8-week	Single injection	0.3 mg/kg	9	3	88.9	\	20-49
Nemoto, 2016b (not available)	Japan	1/1b	8-week	Single injection	1.0 mg/kg	9	3	66.7	\	20-49
Nemoto, 2016c (not available)	Japan	1/1b	8-week	Single injection	3.0 mg/kg	9	3	77.8	\	20-49
Ruzicka, 2017a (NCT01986933)	Multinational	2	12-week	3 Q4W	0.1 mg/kg	53	18	53	\	18-65
Ruzicka, 2017b (NCT01986933)	Multinational	2	12-week	3 Q4W	0.5 mg/kg	54	18	41	\	18-65
Ruzicka, 2017c (NCT01986933)	Multinational	2	12-week	3 Q4W	2.0 mg/kg	52	17	60	\	18-65
Silverberg, 2020a (NCT03100344)	Multinational	2b	24-week	4 Q4W	0.136 mg/kg	55	19	52.7	54.4	18+
Silverberg, 2020b (NCT03100344)	Multinational	2b	24-week	4 Q4W	0.390 mg/kg	55	19	50.9	54.4	18+
Silverberg, 2020c (NCT03100344)	Multinational	2b	24-week	4 Q4W	1.118 mg/kg	55	18	45.6	54.4	18+
Stander, 2020 (NCT03181503)*	Multinational	2	18-week	3 Q4W	0.5 mg/kg	34	36	44	39	18+

*These studies were conducted among non-AD patients.

There was a mistake in **Table 2** as published. We omitted a subgroup analysis for patients with AD only; this has now been added to the second row after ‘Pruritus VAS”.

The corrected **Table 2** appears below.

**Table 2:** Combined results of the subgroup analyses.

Variables	N	WMD (95%CI)	P (Heterogeneity)	I-square, %	P
Pruritus VAS	14	-18.86 (-27.57, -10.15)	0.005	56.2	<0.001
Pruritus VAS in AD	10	-26.05 (-32.63, -19.46)	0.804	0	<0.001
Single injection	6	-3.76 (-10.36, 2.84)	0.836	0	0.264
3 injections	4	-34.35 (-45.77, -22.94)	0.572	0	<0.001
4 injections	4	-22.22 (-30.33, -14.10)	0.946	0	<0.001
EASI	7	-11.76 (-2 0.55, -2.96)	0.978	0	0.009
3 injections	3	-8.75 (-26.72, 9.22)	0.630	0	0.340
4 injections	4	-12.71 (-22.79, -2.62)	0.991	0	0.014
Variables	N	RR (95%CI)	P (Heterogeneity)	I-square, %	P
AE	11	1.03 (0.93, 1.13)	0.980	0	0.593
Single injection	3	0.84 (0.63, 1.13)	0.836	0	0.249
3 injections	4	1.04 (0.88, 1.25)	0.983	0	0.626
4 injections	4	1.05 (0.93, 1.18)	0.898	0	0.415

There were few textual/grammatical errors throughout the article as given below:

In the **Abstract**; the phrase “in treating patients with AD” has been corrected to: “in treating patients with pruritus”

In Section “1. **Introduction** Section, Paragraph Number: 3”; “pruritus” was incorrectly written as “AD”. The phrase now reads: “pruritus by assessing the amelioration of pruritus”.

In Section “2. **Materials and Methods** Section, 2.1 *“Literature search”* Sub-section, Paragraph Number: 1”; “and”, has been corrected to “or”. The phrase now reads: “atopic dermatitis or pruritus”.

In Section “**Results** Section, 3.2 *“Characteristics of the studies”*, Paragraph Number: 1”; there was a lack of the explanation of the two no-AD studies. This has been added now and it reads: “Two of the included studies did not involve AD patients, one focused on individuals with prurigo nodularis (Ständer, S., et al., 2020), and the other on hemodialysis (Kinugasa, E., 2021).”

The original version of this article has been updated.

