# Supplementary figures and images for: Safety and Efficacy of Nemolizumab for Patients with Pruritus: A Systematic Review and Meta-Regression Analysis of Randomized Controlled Trial
Source: Front Immunol. 2022 Apr 26;13:825312. doi: 10.3389/fimmu.2022.825312 (PMC9086972; doi:10.3389/fimmu.2022.825312)

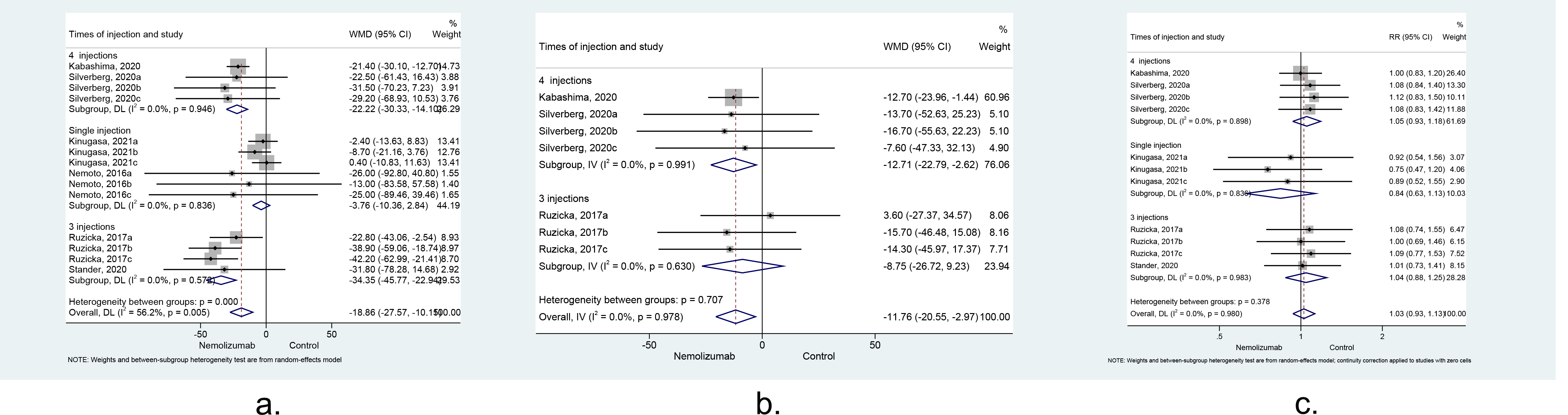

Supplement: Supplementary Figure 1 — (A) Forest plot of pruritus visual analog scale comparing nemolizumab and placebo by the frequency of injection. (B) Forest plot of Eczema Area and Severity Index comparing nemolizumab and placebo by the frequency of injection. (C) Forest plot of adverse events comparing nemolizumab and placebo by the frequency of injection. [file Image_1.tif]

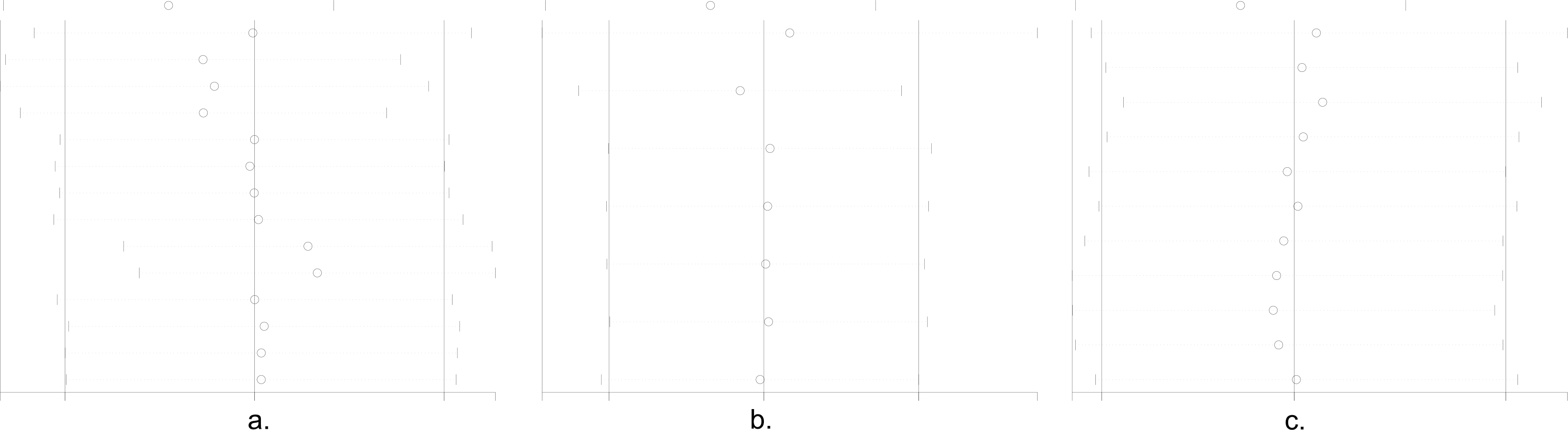

Supplement: Supplementary Figure 2 — (A) Sensitivity analysis of pruritus visual analog scale. (B) Sensitivity analysis of Eczema Area and Severity Index. (C) Sensitivity analysis of adverse events [file Image_2.tif]

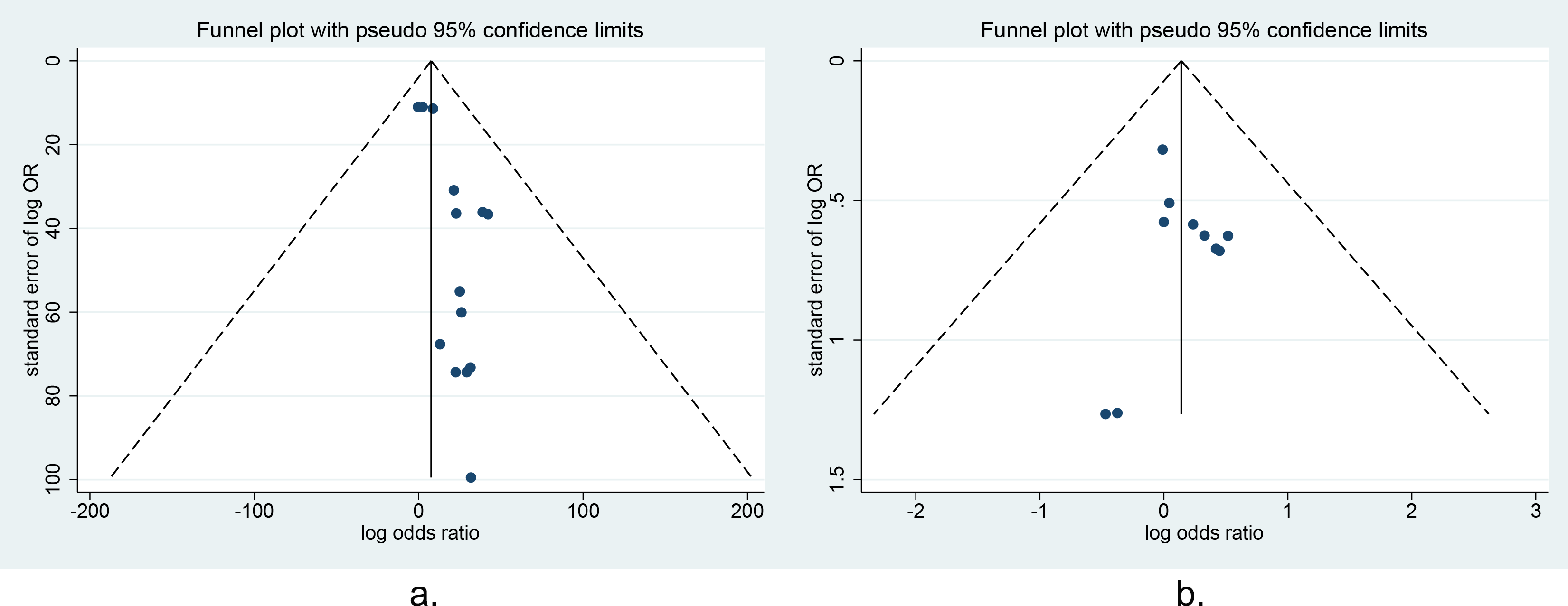

Supplement: Supplementary Figure 3 — (A) Funnel plots for pruritus visual analog scale. (B) Funnel plots for adverse events. [file Image_3.tif]
