# Supplementary material for: Safety and Efficacy of Nemolizumab for Patients with Pruritus: A Systematic Review and Meta-Regression Analysis of Randomized Controlled Trial
Source: Front Immunol. 2022 Apr 26;13:825312. doi: 10.3389/fimmu.2022.825312 (PMC9086972; doi:10.3389/fimmu.2022.825312)
Supplement: Supplementary file 4 [file Table_1.docx]

| Supplementary Table 1. Cochrane risk of bias assessment tool 2 (ROB-2) for randomized controlled trial | | | | | | |
| --- | --- | --- | --- | --- | --- | --- |
| Study | 1 | 2 | 3 | 4 | 5 | 6 |
| Kabashima, 2020 | Low | Low | Low | Low | Low | Low |
| Kinugasa, 2021 | Some concerns | Low | Low | Low | Low | Low |
| Nemoto, 2016 | Low | Low | Low | Low | Low | Low |
| Ruzicka, 2017 | Low | Low | Low | Low | Low | Low |
| Silverberg, 2020a | Some concerns | Low | Low | Low | Low | Low |
| Stander, 2020 | Low | Low | Low | Low | Low | Low |
| 1.Bias arising from the randomization process; 2. Bias due to deviations from intended interventions; 3. Bias due to missing outcome data; 4. Bias in measurement of the outcome; 5. Bias in selection of the reported result; 6. Overall bias. | | | | | | |
