# Supplementary material for: Safety and Efficacy of Nemolizumab for Patients with Pruritus: A Systematic Review and Meta-Regression Analysis of Randomized Controlled Trial
Source: Front Immunol. 2022 Apr 26;13:825312. doi: 10.3389/fimmu.2022.825312 (PMC9086972; doi:10.3389/fimmu.2022.825312)
Supplement: Supplementary file 5 [file Table_2.docx]

Supplementary Table 2. Information of the common adverse events.

| Study | Gastrointestinal disorders | General disorders and administration site conditions | Infections and infestations | Nasopharyngitis | Skin and subcutaneous tissue disorders | Dermatitis atopic | Respiratory, thoracic & mediastinal disorders |
| --- | --- | --- | --- | --- | --- | --- | --- |
| Kabashima, 2020 | 14 (9.8) | 20 (14.0) | 41 (28.7) | 18 (12.6) | 46 (32.2) | 33 (23.1) | \ |
| Kinugasa, 2021a | \ | \ | \ | 4 (26.7) | \ | \ | \ |
| Kinugasa, 2021b | \ | \ | \ | 2 (15.4) | \ | \ | \ |
| Kinugasa, 2021c | \ | \ | \ | 2 (14.3) | \ | \ | \ |
| Ruzicka, 2017a | \ | \ | \ | 9 (17.0) | \ | 11 (21.0) | \ |
| Ruzicka, 2017b | \ | \ | \ | 6 (11.0) | \ | 10 (19.0) | \ |
| Ruzicka, 2017c | \ | \ | \ | 5 (10.0) | \ | 11 (21.0) | \ |
| Silverberg, 2020a | \ | \ | 34 (61.8) | 18 (32.7) | 18 (32.7) | 12 (21.8) | 6 (10.9) |
| Silverberg, 2020b | \ | \ | 34 (59.6) | 14 (24.6) | 23 (40.4) | 14 (24.6) | 13 (22.8) |
| Silverberg, 2020c | \ | \ | 34 (59.6) | 13 (22.8) | 23 (40.4) | 16 (28.1) | 12 (21.1) |
| Stander, 2020 | 7 (21.0) | 5 (15.0) | 10 (29.0) | 5 (15.0) | 10 (29.0) | 5 (15.0) | 0 (0) |
