# Supplementary material for: Safety and Efficacy of Nemolizumab for Patients with Pruritus: A Systematic Review and Meta-Regression Analysis of Randomized Controlled Trial
Source: Front Immunol. 2022 Apr 26;13:825312. doi: 10.3389/fimmu.2022.825312 (PMC9086972; doi:10.3389/fimmu.2022.825312)
Supplement: Supplementary file 6 [file Table_3.docx]

Supplementary Table 3. Publication bias

|  | Begg’s test | | Egger’s test | |
| --- | --- | --- | --- | --- |
|  | Z | P | T | P |
| Pruritus VAS | -0.05 | >0.999 | -0.58 | 0.574 |
| AE | 0.72 | 0.474 | 0.39 | 0.709 |
