# Supplementary material for: Safety and Efficacy of Nemolizumab for Patients with Pruritus: A Systematic Review and Meta-Regression Analysis of Randomized Controlled Trial
Source: Front Immunol. 2022 Apr 26;13:825312. doi: 10.3389/fimmu.2022.825312 (PMC9086972; doi:10.3389/fimmu.2022.825312)
Supplement: Supplementary file 7 [file Table_4.docx]

Supplementary Table 4. Meta-regression

|  | Univariate | | |
| --- | --- | --- | --- |
|  | Coefficient | 95%CI | P |
| Pruritus VAS |  |  |  |
| Dose | -0.61 | -15.77, 14.54 | 0.931 |
| Study duration | 2.04 | -1.13, 5.20 | 0.186 |
| EASI |  |  |  |
| Dose | 3.04 | -63.78, 69.86 | 0.911 |
| Study duration | 0.31 | -11.14, 11.77 | 0.948 |
| AE |  |  |  |
| Dose | -0.10 | -0.62, 0.41 | 0.657 |
| Study duration | 0.03 | -0.05, 0.11 | 0.405 |
